# The Zinc Finger SET Domain Gene *Prdm14* Is Overexpressed in Lymphoblastic Lymphomas with Retroviral Insertions at *Evi32*

**DOI:** 10.1371/journal.pone.0003823

**Published:** 2008-11-27

**Authors:** E. J. Dettman, Monica J. Justice

**Affiliations:** 1 Department of Molecular and Human Genetics, Baylor College of Medicine, Houston, Texas, United States of America; 2 Department of Molecular Physiology and Biophysics, Baylor College of Medicine, Houston, Texas, United States of America; Deutsches Krebsforschungszentrum, Germany

## Abstract

**Background:**

AKXD recombinant inbred strains of mice have proven to be very useful in the identification of potential oncogenes and tumor suppressors involved in the development of lymphoid and myeloid malignancies. In these tumors, the hematopoietic insertion of an active AKV murine leukemia virus (MuLV) is associated with the onset of disease. Common sites of retroviral insertion (CIS) identify genes causally associated with the development or initiation of lymphoma.

**Methodology:**

In the present study, we analyzed a previously uncharacterized CIS, Ecotropic Viral Integration Site 32 (*Evi32),* which is located on mouse chromosome 1. We analyzed candidate genes in the region to identify those involved in *Evi32* mediated oncogenesis.

**Results:**

Here we show that proviral insertion at *Evi32* correlates with significant overexpression of a putative transcription factor, PR-domain containing 14 (*Prdm14*). Tumors with insertions at *Evi32* are consistently lymphoid in nature. *Prdm14* is normally expressed early in embryonic development with the highest expression in undifferentiated embryonic stem (ES) cells. This study implicates *Prdm14* as a proto-oncogene involved in lymphoblastic lymphoma formation.

## Introduction

Retroviral mutagenesis is an invaluable tool for the identification of novel genes involved in the development and progression of cancer in mice. Germ line retroviruses in certain inbred mouse strains replicate and infect other cells within the hematopoietic system. Integrated proviruses may alter the expression of nearby genes, because the long terminal repeat (LTR) elements contain enhancers and promoters of transcription [1]. If a proviral integration alters gene expression in such a way that it gives a growth advantage to host cells, a clonal expansion of those cells can occur, ultimately leading to tumor formation. Somatic proviral insertions contained in tumor tissue act as a molecular tag for the identification of genomic loci containing genes with oncogenic transformation ability [2]. In this study, several AKXD recombinant inbred mouse strains were examined that harbor endogenous replication competent proviruses in their germline and have a high incidence of lymphoma formation [3]. Each AKXD strain has variable susceptibility to lymphoma formation affecting both cell type and frequency [4].

Since it is highly unlikely that a proviral integration would occur in the same region multiple times in the absence of oncogenic selection, CISs are assembled to eliminate non-pathogenic insertions. One CIS that was identified in multiple retroviral insertional mutagenesis cancer screens is *Evi32* [3], [5], [6], [7]. In previous work, *Evi32* was believed to affect flanking gene nuclear receptor coactivator 2/transcription intermediary factor 2 (*NCoA2/Tif2)* [5], [6], [7]. Here we show that a putative transcription factor, *Prdm14*, is overexpressed in tumors with proviral integration at *Evi32*. *Prdm14* is normally expressed at low levels in most control tissues tested but is massively upregulated in all *Evi32* tumors. Prdm14 contains a SET domain and six zinc finger repeats. SET domains are contained in a variety of transcription factors and have been shown to be responsible for methylation of histones [8]. Interestingly, a large proportion of genes containing SET domains have been implicated in leukemia/lymphoma, such as the mixed-lineage leukemia (Mll) proteins and B Lymphocyte-induced Maturation Protein 1 (*Blimp1*) suggesting that this domain is commonly involved in tumorigenesis [9]. Our research suggests that *Prdm14* is a novel proto-oncogene involved in the formation of lymphoma in mice.

## Results

### Evi32

**Figure 1:**
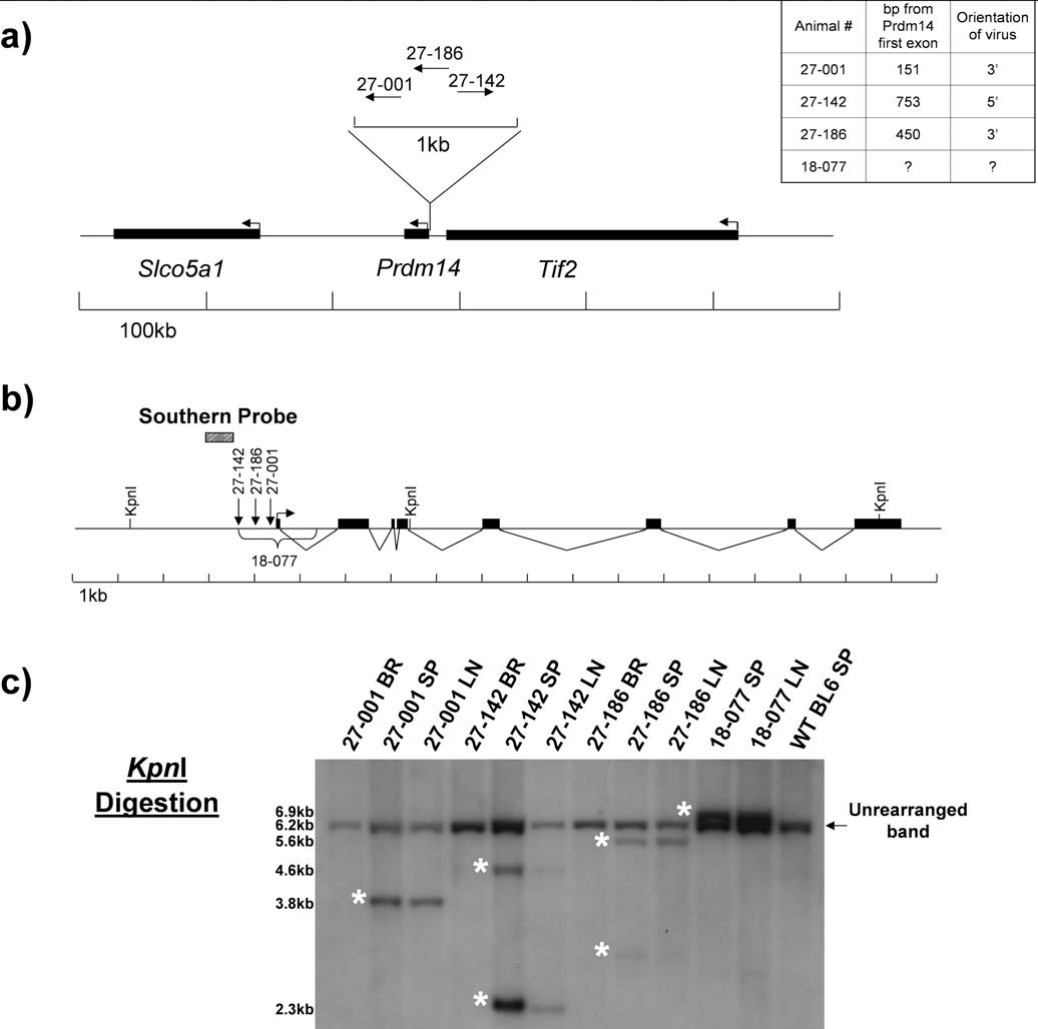
*Evi32* Viral Insertion Sites. 1a) The genomic locus surrounding *Evi32* insertions. Locations of proviral insertions were determined by either VISA PCR or by PCR after identification of *Evi32* integrations. Arrows indicate direction of transcription and the orientation of the retroviral integration. The exact orientation and location of the retroviral integration in animal 18-077 was not determined, but it is contained between the *Hind*III and *Xba*I digestion sites as determined by Southern blot analysis. Table at top right indicates the distance of each retrovirus from the first exon of *Prdm14*. 1b) Closeup view of *Evi32* integrations. Restriction enzymes indicated were those used in digestion of tumor DNA to show genomic rearrangements at *Evi32*. Arrow indicates direction of transcription of *Prdm14*, and the black boxes show the eight exons of *Prdm14*. *Prdm14* gene structure is based on VEGA prediction (OTTMUSG00000017097) obtained from the Ensembl database. RT-PCR amplification indicates that the VEGA prediction is a true *Prdm14* transcript. 1c) Southern blots showing retroviral integrations at *Evi32*. Genomic DNA from indicated tumors was digested with *Kpn*I. Brain serves as somatic tissue control from same animal to show rearrangement of *Evi32* locus within the tumor tissue only. Rearranged bands seen in *Evi32* tumors are indicated by asterisks and show genomic rearrangements at *Evi32*. BR = Brain, SP = Spleen, LN = Lymph Node.

VISA PCR identified two tumors from mice 27-001 and 27-142 (27 indicates AKXD strain 27 followed by the mouse number) with insertions at *Evi32* [3]. *Evi32* is located between *Tif2* and *Prdm14* on mouse chromosome 1 (Figure 1a). Both insertion sites are located very close to the start site of *Prdm14*, 27-001 is 151bp from the first exon, and 27-142 is 753 bp. Both insertions are located approximately 15 kb from *Tif2*, and 136 kb from solute carrier organic anion transporter family member 5A1 (*Slco5a1)*. The provirus in 27-001 inserted in the same orientation as *Prdm14*, and 27-142 inserted in the opposite direction. Southern blot analysis after *Kpn*I digestion showed that unique tumor specific bands were present in both *Evi32* tumors, indicating the presence of somatically acquired proviral integrations at the locus (Figure 1c). The intensity of the rearranged band indicates that the integrations at *Evi32* are monoclonal. Digestion with *EcoR*V and *Xba*I also confirmed somatically acquired proviral rearrangements at *Evi32* (data not shown).

To identify additional *Evi32* tumors, all of our AKXD tumors (from 204 animals) were further screened by Southern blotting using *Eco*RV digestion. This analysis revealed that tumors from mice 27-186 and 18-077 (from AKXD strain 18, mouse 77) contain rearrangements at *Evi32* (Figure 1c). Digestion of 18-077 and 27-186 genomic DNA with *Eco*RI indicates that both of these proviruses are ecotropic (data not shown). The retroviral insertion in tumor 27-186 is located approximately 450 bp from the first exon of *Prdm14* between the 27-001 and 27-142 insertions and integrated in the same orientation as *Prdm14* (Figure 1b). The genomic location of this insertion was determined using PCR amplification (data not shown). The exact location of the insertion in the tumors obtained from mouse 18-077 was not determined, but it is located within the 1.4 kb region (as determined by Southern blot analysis with *Hind*III and *Xba*I) surrounding the first exon of *Prdm14* indicated in Figure 1b. We were unable to amplify the virus using viral specific primers, which could be due to retroviral rearrangements that prevented PCR amplification from the virus. Southern blot analysis after *Kpn*I digestion indicates that the LTR elements may have been deleted in 18-077, making precise localization difficult. Analysis of the number of monoclonal ecotropic somatic retroviral integrations in the tumors indicated a relatively low number of integrations (Supplementary Figure S1 and Table S1). Southern analysis of tumors obtained from 27-186 and 18-077 indicated just a single retroviral integration event. Tumors from 27-001 and 27-142 had three and two somatic insertions respectively.

### *Tif2* and *Slco5a1* are not misexpressed in tumors containing insertions at *Evi32*

**Figure 2:**
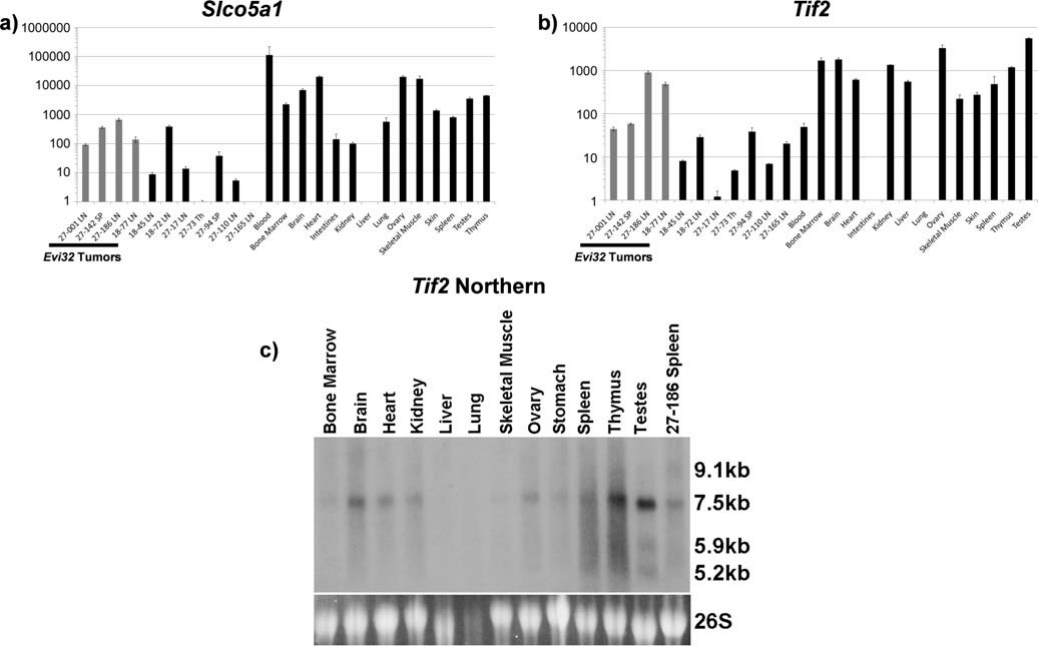
*Tif2* and *Slco5a1* are not misexpressed in *Evi32* tumors. 2a) Q RT-PCR was performed on cDNA prepared from total RNA samples from tumors with and without insertions at *Evi32* and control tissue obtained from 8 week old C57BL/6J mice. Expression of *Slco5a1* was normalized to 18S expression levels and then quantified. We found that the expression of *Slco5a1* does not appear to be consistently altered with *Evi32* proviral insertions and is expressed at lower levels than seen in normal spleen, thymus, and bone marrow. Expression levels are shown on a log scale to show lower levels of expression. 2b) Expression of *Tif2* was quantified in the same manner as *Slco5a1*. This analysis revealed that *Tif2* is expressed at similar or lower levels than normal spleen, thymus, or bone marrow in all *Evi32* tumors. Expression levels are shown on a log scale to show lower levels of expression. 2c) Northern blot analysis of *Tif2* was performed using total RNA obtained from *Evi32* tumor 27-186 and control tissue obtained from 8 week old C57BL/6J mice. The probe to *Tif2* was directed towards common exons found in all isoforms. Sizes were estimated using 18S and 26S migration distances. The 27-186 *Evi32* tumor showed the greatest expression of *Tif2* as determined by Q RT-PCR, but shows no increase in expression by northern analysis. SP = Spleen, LN = Lymph Node, Th = Thymus.

Quantitative RT-PCR (Q RT-PCR) was used to determine the level of expression of the genes surrounding *Evi32*. Since retroviruses can alter expression of genes up to 200 kb away [10], we analyzed expression of *Slco5a1* in tumors with *Evi32* proviral integrations (Figure 2a). We found that *Slco5a1* was expressed in all Evi32 tumors at lower levels than seen in normal spleen, thymus, and bone marrow. Lower levels of expression of *Slco5a1* were also observed in all tumors that lacked proviral insertions at *Evi32*. Therefore, expression of *Slco5a1* is not significantly altered by proviral integration at *Evi32*.

Q RT-PCR was also used to determine the level of expression of *Tif2* (Figure 2b), which is listed in the Retrovirus Tagged Cancer Gene Database (RTCGD) as the affected putative oncogene associated with viral insertion in this region (http://rtcgd.abcc.ncifcrf.gov/). All *Evi32* tumors expressed *Tif2* at similar or lower levels than normal hematopoietic tissues (spleen, thymus, and bone marrow). Additionally, the level of *Tif2* expression by northern blot analysis in tumor 27-186 appeared to be the same as control spleen (Figure 2c). Therefore, no consistent alteration in the expression of *Tif2* could be correlated with proviral insertion at *Evi32*.

*TIF2* is capable of inducing acute myeloid leukemia as a gene fusion product with monocytic leukemia zinc finger (*MOZ)*. However, expression of unaltered *TIF2* alone is unable to induce leukemia formation [11]. To rule out any rearrangements that may result in aberrant transcripts of *Tif2,* we performed northern blot analysis. Three isoforms of *Tif2* are annotated in the Ensembl database, and a probe consisting of eight common exons was used to detect *Tif2* rearrangements. This analysis revealed no altered gene products of *Tif2* in the *Evi32* tumor 27-186 (Figure 2c). Furthermore, all bands were consistent with previously described *Tif2* transcript sizes [12], [13], [14]. All other tumors were also analyzed by northern analysis and no additional transcripts were revealed (data not shown). Together, these analyses indicate that the transcription of *Tif2* is not altered with proviral integration at *Evi32*.

### *Prdm14* is overexpressed in *Evi32* tumors

*Prdm14* is encoded by eight exons spanning 13.7 kb on mouse chromosome 1. The intron-exon structure of the gene is well conserved with the human homologue *PRDM14* on human chromosome 8. The predicted protein of Prdm14 is that of a transcription factor, containing six C2H2 type zinc finger domains and a SET domain. Other proteins containing these domains have been shown to have a role in transcription, such as Blimp1 (Reviewed in [15]).

**Figure 3:**
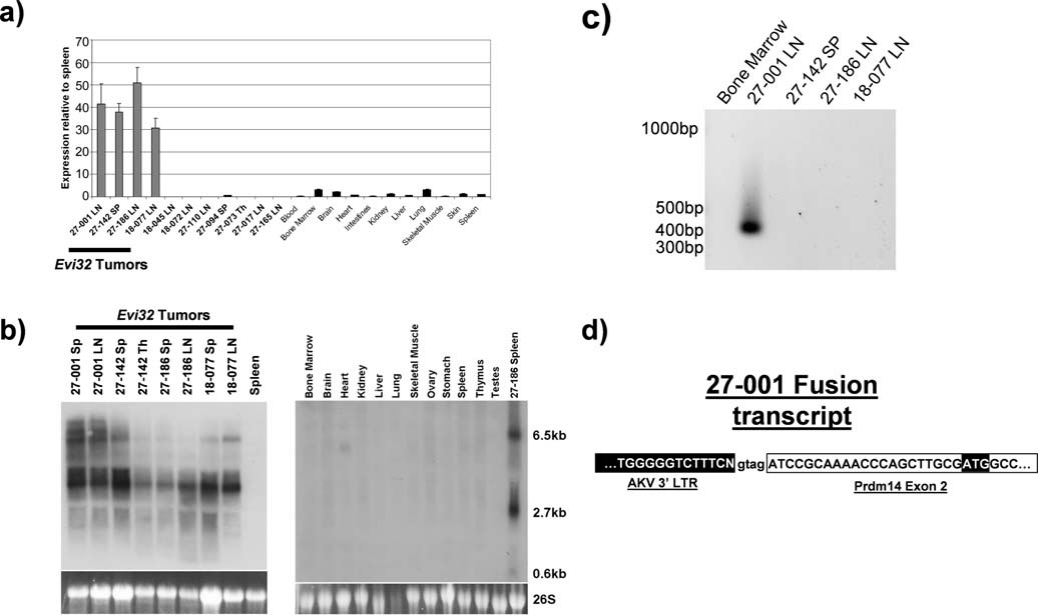
*Prdm14* is overexpressed in *Evi32* tumors. 3a) Q RT-PCR was performed on cDNA prepared from total RNA samples from AKXD tumors with and without insertions at *Evi32* and control tissue obtained from 8 week old C57BL/6J mice. As before, expression levels were normalized to 18S expression then quantified. This analysis revealed that *Prdm14* expression was upregulated in all *Evi32* tumors. The expression levels of *Prdm14* in *Evi32* tumors varied between 30 and 50 fold more than in control spleen. 3b) Northern analysis of *Prdm14* was performed using total RNA obtained from *Evi32* tumors and control tissue obtained from 8 week old C57BL/6J mice. The probe to *Prdm14* was nearly the full length coding sequence amplified by PCR. Sizes were estimated using 18S and 26S migration distances. This analysis shows specific upregulation of *Prdm14* in all *Evi32* tumors. 3c) RT-PCR was performed on *Evi32* tumors using a viral 3′ LTR specific primer coupled with a *Prdm14* second exon primer. cDNA obtained from the 27-001 produced a prominent band consistent with a viral fusion transcript. 3d) The amplicon from 27-001 was sequenced, and viral elements were found to be linked to the second exon of *Prdm14* which contains the beginning of the coding sequence of *Prdm14*. SP = Spleen, LN = Lymph Node, Th = Thymus.

Q RT-PCR was performed on the *Evi32* tumors using the VEGA predicted sequence for *Prdm14* as a guide. We found that this transcript was massively upregulated in all tumors containing *Evi32* insertions compared to all *Evi32* negative AKXD tumors and control tissue examined (Figure 3a). *Prdm14* was expressed between 30 and 50 fold more in *Evi32* tumors than in control spleen. Moreover, this overexpression was seen in all *Evi32* tumors and observed only in those tumors with proviral insertions at *Evi32*. This shows that overexpression of *Prdm14* is limited to AKXD tumors containing *Evi32* proviral integrations.

Northern analysis was then performed to confirm the overexpression of *Prdm14* and determine the size of the *Prdm14* transcripts. This analysis showed a high level of expression of *Prdm14* specifically in all *Evi32* tumors, but not in any control RNA tested (Figure 3b). Two major transcripts were observed in the tumors: one 2.7 kb and one 6.5 kb. The more prominent 2.7 kb transcript is consistent with the VEGA prediction OTTMUSG00000017097 of *Prdm14* (http://vega.sanger.ac.uk/). This transcript is also supported by RT-PCR analysis using RNA obtained from tumor tissue (data not shown). The larger transcript could be a pre-mRNA of *Prdm14* or the result of altered splicing due to retroviral integration at *Evi32*. Tumor 27-001 also appeared to have an additional band, which was slightly larger than the 6.5 kb band. However, this additional transcript was not observed in any of the other tumors.

To determine whether transcription of *Prdm14* was being driven directly from the proviral LTR elements, we performed RT-PCR using viral 3′ LTR specific primers [16]. A primer specific to the second exon of *Prdm14* (first coding exon) was used as the reverse primer. All *Evi32* tumors were analyzed; however, only 27-001 produced a PCR product (Figure 3c). The amplicon was sequenced and showed a fusion between the second exon of *Prdm14* and the 3′ LTR of the provirus (Figure 3d). This viral fusion transcript could be the additional larger transcript seen in the northern analysis of 27-001 in Figure 3c. The presence of this additional message suggests that transcription of *Prdm14* is being driven from the 3′ LTR of the provirus and splicing into *Prdm14*, further suggesting that *Prdm14* is being altered specifically by proviral integrations at *Evi32*.

### Normal expression of *Prdm14*

**Figure 4:**
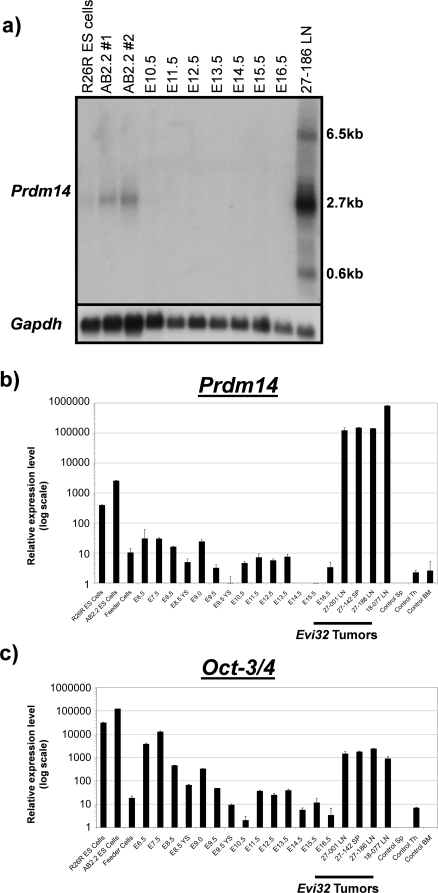
*Prdm14* is expressed most highly in ES cells. 4a) Northern analysis of embryonic tissue compared with the *Evi32* tumor 27-186. *Gapdh* was used as a loading/RNA integrity control. We found expression of the 2.7 kb band in all ES cell samples, but none in any of the other embryonic tissues. The larger 6.5 kb transcript seen in the *Evi32* tumor is not found in the ES cells. 4b and c) Quantitative RT-PCR was performed on cDNA obtained from the same tissue from 4a. *Prdm14* and *Oct-3/4* expression levels were quantified relative to 18S expression levels within each tissue. Expression levels are on a log scale to emphasize lower levels of expression. Results show specific expression of *Prdm14* within *Oct-3/4* positive ES-cells. All embryonic days are whole embryos unless otherwise indicated. YS = yolk sac.

Since very little is known about *Prdm14*, we sought to determine its normal expression pattern. Compared to *Evi32* tumors, we found very low levels of expression of *Prdm14* in all normal adult tissues as determined by Q RT-PCR (Figure 3a, 3b). We next examined expression of *Prdm14* during embryonic development. The highest embryonic levels of expression were found in undifferentiated embryonic stem cells in culture (AB2.2 and Rosa 26 reporter ES cells) (Figure 4b). All other embryonic time points tested showed relatively low levels of *Prdm14*; however, amongst these, the earlier embryonic stages (starting at E6.5) show higher levels of expression of *Prdm14* than do later developmental time points (up to E16.5). The 2.7 kb transcript is present in ES cells, but the 6.5 kb band is absent (Figure 4a). To confirm that the ES cells were in an undifferentiated state, *Oct-3/4* was used as a marker of pluripotency [17]. We found high levels of *Oct-3/4* expression in the ES cells, with low expression levels in all other embryonic tissues tested (Figure 4c). This analysis also revealed that *Oct-3/4* is expressed at relatively high levels in *Evi32* tumors, which was striking because we found no expression of *Oct-3/4* in control bone marrow and spleen. We examined AKXD tumors without proviral insertions at *Evi32* from the AKXD-27 and -18 strains, in which all *Evi32* insertions occurred, as well as from the AKXD-9, -10, -13, and -16 strains, which did not have any *Evi32* insertions, for *Oct-3/4* expression. Although some strains appeared to have lower expression, there was not significantly more *Oct3/4* expression in *Evi32* tumors compared with those lacking *Evi32* integrations (p = 0.53 by the student's t-test) (Supplementary Figure S2).

### Analysis of *Evi32* tumors

*Evi32* tumors were originally typed by histopathology and B- and T-cell receptor rearrangements, and were classified as lymphoblastic lymphomas (Supplementary Table S1). Altogether, insertions at *Evi32* occurred in 2.0% of the 204 AKXD tumors acquired in this study. However, within AKXD strains 18 and 27, *Evi32* integrations occurred in 2.4% (1 of 42 tumors) and 7.3% (3 of 41 tumors) of all tumors, respectively. All three tumors from AKXD strain 27 (27-001, 27-142, and 27-186) were classified as mixed B/T cell by Southern analysis. In total, *Evi32* insertions occurred in 13% (3 of 23 total) of the mixed B/T lineage tumors and in 43% (3 of 7 tumors) of the mixed B/T tumors from AKXD-27. The fourth tumor (18-077) was classified as a T-Cell lymphoblastic lymphoma in AKXD strain 18. No *Evi32* insertions were identified in 121 tumors from seven other AKXD lines (2, 3, 9, 10, 13, 14, and 16). However, *Evi32* retroviral integrations were found in six additional tumors in the Mouse RTCGD. Four of these tumors were classified as B-cell, one was a mixed B-/T-cell, and the last was a myeloid tumor on the BXH2-*Runx1*+/− background).

## Discussion

In this work, we have demonstrated that *Evi32* is a CIS in AKXD mice. All insertions were clustered very tightly at the start of the SET domain-containing gene, *Prdm14*. Previously, it was thought that insertions at this CIS would affect *Tif2*, since human *TIF2* acts as a fusion oncogene with *MOZ* [18], [19]. When this fusion oncogene is overexpressed in mice via retroviral transduction, the mice consistently develop acute myeloid leukemia [11], whereas tumors with insertions at *Evi32* are lymphoblastic lymphomas. Furthermore, we have found that *Tif2* transcripts are not altered in tumors with insertions at *Evi32*. Instead, each of the tumors with insertions at *Evi32* expresses *Tif2* at a level that is similar to or lower than normal hematopoietic organs. However, in all *Evi32* tumors, *Prdm14* is highly overexpressed. Furthermore, in one *Evi32* tumor, expression of *Prdm14* is driven from the LTR elements of the integrated provirus. Proviral-gene fusions have been observed at other oncogenes, which are activated by the insertion, including *Evi3* [16]. The chance that a retrovirus would integrate in such a manner as to allow expression of *Prdm14* directly from the LTR elements is unlikely without this event contributing to tumor formation. Therefore, we propose that retroviral integration at *Evi32* leads to a massive overexpression of *Prdm14* contributing to lymphoma in these mice.

There is substantial evidence that *PRDM14* is involved in human cancer as well. In a screen for differentially methylated loci in human breast cancer epithelial cells, the second intron of *PRDM14* had increased levels of DNA methylation [20]. Higher levels of *PRDM14* expression correlated with the increased methylation state in those breast cancer cells [20]. Furthermore, *PRDM14* is overexpressed in 57–75% of breast cancers examined and is correlated with genomic amplification at its locus indicating a causative role [21]. Our research indicates that *Prdm14* may also be involved in murine leukemia/lymphoma development.

Another PR-domain containing gene, *PRDM16*, has also been implicated in t(1;3)(p36;q21) and t(1;21)(p36;q22) acute myeloid leukemias [22], [23], [24], [25]. In these cases, the short form lacking the PR-domain, *MEL1S/sPRDM16,* is often overexpressed [26], [27]. Additionally, retroviral integrations in *Prdm16* in mouse hematopoietic cells has been associated with myeloid leukemia, as well as the immortalization of progenitor cells [28], [29]. A recent paper implies that the oncogenic potential of PRDM16 lies in sPRDM16, which lacks the PR-domain [30]. However, for *Prdm14*, we observed high expression of a PR-domain containing transcript in all *Evi32* lymphomas. This could indicate that among PRDM family members, the lack of the PR-domain is not always required for transformation.

Within our screen, all tumors with insertions at *Evi32* occurred in mice from only two AKXD lines of nine total strains aged [3]. Furthermore, three of four of the *Evi32* tumors were identified from AKXD strain 27. This is probably due to strain specific genetic background differences in strain 27 that predispose mice to *Prdm14*-induced lymphoid cancer. Therefore, additional genes in AKXD-27 may cooperate with *Prdm14*. Identifying these cooperating genes may uncover mechanisms by which *Evi32* tumorigenesis occurs. This illustrates the importance of using multiple genetic backgrounds in retroviral mutagenesis screens. If AKXD-27 had not been included, *Evi32* would not have been identified as a common insertion site in our study. However, it was identified in other screens and in a recent screen in a Bloom Syndrome deficient genetic background [3], [5], [6], [7]. Such information suggests that *Prdm14* may predispose to leukemia development only under certain genetic conditions.

Very recently, mice deficient for *Prdm14* were generated [31]. While the mice appeared normal; they had impaired fertility. The *Prdm14^−/−^* mice failed to develop embryonic germ cells due to a defect in establishment of a pluripotent state and a failure to reset the epigenetic programming in primordial germ cells. These two events are necessary for the formation of embryonic germ cells. Interestingly, expression of the pluripotency marker *Oct-3/4* was observed in all AKXD tumors examined here, allowing for the possibility that pluripotency may play a role in tumor susceptibility when Prdm14 is overexpressed. Together, these observations suggest that the function of *Prdm14* in tumor development may be promoted in cells with a less differentiated phenotype. It has also been shown that the ES cell pluripotency markers OCT4, SOX2, and NANOG bind the promoter of *PRDM14* in human ES cells [32]. si-RNA knockdown of any of these three genes results in a reduction of *PRDM14* in human embryonic carcinoma cells [33]. This implies that Prdm14 may be very important in the maintenance of an undifferentiated state since the three major ES cell genes appear to regulate Prdm14 expression in some manner. Unfortunately, the transcriptional targets of Prdm14 remain to be determined; finding these may further elucidate the direct role of Prdm14 in cancer development.

This work shows that despite advances in transposable element mutagenesis [34], [35]; retroviral mutagenesis screens still represent a valuable resource for the identification of novel proto-oncogenes involved in leukemogenesis. It also highlights the importance of studying low insertion frequency CISs. Despite *Evi32* being identified as a CIS in other work, *Prdm14* went unnoticed, likely because of the low numbers of tumors with insertions at this locus. Examination of other genes at CISs that have not been studied could reveal other novel cancer genes relevant to human cancer. Further work on Prdm14 will provide a greater knowledge of the role of SET domain genes in cancer development, as well as a direct link between tumorigenesis and ES cell biology.

## Materials and Methods

### AKXD Tumor Screen and VISA PCR

All experiments involving mice were carried out under the approval of the Institutional Animal Care and Use Committee at Baylor College of Medicine (BCM). Mice are housed in the Transgenic Mouse Facility at BCM, under the care of the Center for Comparative Medicine, which is accredited by the Association for Assessment and Accreditation of Laboratory Animal Care International.

Two hundred and four animals from AKXD mouse strains 2, 3, 9, 10, 13, 14, 16, 18, and 27 developed leukemia or lymphoma. Tumors were named first by AKXD strain followed by the animal number, and were classified by histopathology and molecular analysis of B and T-cell rearrangements as previously described [3]. VISA (Viral Insertion Site Amplification) PCR was performed on tumor DNA to identify flanking genomic DNA regions as previously described [3], [36]. BLAST analysis of viral sequence tags (VSTs) was performed to align proviral integrations with the mouse genome. CISs were assembled when multiple VSTs hit a region of less than 25 kb. Tumor information can be found on www.mouse-genome.bcm.tmc.edu/VISION.

### Southern blot analysis of *Evi32* tumors

DNA was digested with *Eco*RV, *Kpn*I, and *Xba*I. The locations of restriction enzyme sites at the *Evi32* locus based on the published C57BL/6J mouse genome sequence are shown in Figure 1b. A 500 bp probe for *Evi32* was produced by PCR amplification. Location of the probe is indicated in Figure 1b. Primer sets to generate the probe were: Evi32SPF (5′-AGAGCCCTGGATCTCTAGCC-3′) and Evi32SPR (5′-GAGAAAGGGGCGTTTAATCC-3′). The probe to detect retroviral rearrangements was based on pEco, which is directed towards the AKV *env* gene, and is specific for ecotropic proviruses [37]. The probe was amplified from AKXD tissue with pEcoSPF (5′-CATGCACACAATGGAGGATT-3′) and pEcoSPR (5′-GTCTGACAAGACGGGGTTTG-3′). Southern blotting was performed as previously described [38].

### Quantitative RT-PCR (Q RT-PCR)

RNA was produced from snap frozen AKXD tumors and control C57BL/6 tissue using RNA STAT60 (Tel-Test Inc, Friendswood Texas) and extracted by company protocol. cDNA samples were produced from RNA using Multiscribe reverse transcriptase (ABI Systems, Foster City California) with random hexamers. Control tissue was obtained from C57BL/6J mice. Embryonic tissue was obtained after (FVB/N X 129S6/SvEv)F1 female mice were mated with FVB/N male mice and monitored for vaginal plug formation, and then sacrificed at specified embryonic days. Whole embryos were used for RNA preparation. RNA was obtained from ES cell lines AB2.2 and Rosa 26 (R26R), cultured on SNLP 76/7-4 Puro feeder cells. RNA from the feeder cells was also prepared.

Q RT-PCR was performed using the ABI Prism 7000 Sequence Detection System. Primers were designed across exon junctions using Primer Express 3.0 (ABI Systems, Foster City California). Primers to *Tif2* were Tif2RTF (5′-AAGTTCTGAGACgAAGGGTTGGC-3′) and Tif2RTR (5′-GCCATCAGACAAAGAAAAACGATAG-3′), for *Slco5a1* were Slco5a1RTF (5′-ATCCCTTACACAACGGGACCT-3′) and Slco5a1RTR (5′-CCTGTCAGATTCCTATGAGGCAT-3′). Primers for *Prdm14* were Prdm14RTF (5′-CGGAAGGTCTCTGCCTCATG-3′) and Prdm14RTR (5′-CAAAGTGTGGCACATCACCAA-3′). Primers for *Oct-3/4* were Oct3/4F (5′-GCCAATCAGCTTGGGCTAGA-3′) and Oct3/4R (5′-TGGCGCCGGTTACAGAA-3′). 18S amplification was used to normalize the level of cDNA in each samples using primers 18SRTF (5′-GTAACCCGTTGAACCCCATT-3′) and 18SRTR (5′-CCATCCAATCGGTAGTAGCG-3′). All primer sets were optimized by running pairwise combinations of 50 nM, 300 nM, and 900 nM concentrations of forward and reverse primers. SYBR Green 2x master mix (ABI Systems, Foster City California) was used in all reactions. Forty cycles of PCR were used in each Q RT-PCR reaction with a dissociation step to confirm products. All samples were run in triplicate, and error bars represent the standard error of the mean. The relative fold change of gene expression between control and tumor tissue was calculated using the standard 2^−ΔΔCt^ method.

### Northern blot analysis

Total RNA was prepared from snap frozen tissue using RNA-STAT60 (Tel-Test Inc). 10 ug of RNA was electrophoresed on a 1% agarose gel using Northernmax 10x gel denaturing solution (Ambion, Austin TX) in 1x MOPS. Gel was transferred to Hybond N+ membrane (Amersham Biosciences, Piscataway NJ) using an alkaline transfer buffer (3M NaCl, 0.01M NaOH). Membrane was then analyzed using an UltraHyb (Ambion, Austin TX) solution containing 1×10^6^ cpm/ml [α-^32^P]dATP labeled probe , which was a near full-length coding cDNA of *Prdm14* produced by amplification of reverse transcribed cDNA from AKXD tumor 27-001 using primers Prdm14FLF (5′-ATGGCCTTACCGCCCTC-3′) and Prdm14FLR (5′-TTTATGAAGCCTCATGTGGGCAT-3′). BLAST analysis of the 1677 bp *Prdm14* northern probe against murine cDNA reveals limited homology with other genes. The highest conservation is with ENSMUST00000110739 (zinc finger protein 12) with 100% identity across 24 bp, and the next highest is 87% identity with 40 bp of ENSMUST00000108985 (A2BE20_MOUSE). *Tif2* transcripts were analyzed using a probe to eight common exons found in all *Tif2* transcript isoforms, which was PCR amplified using primer sets Tif2NPF (5′-CAGTCCAAGGAGGCATGATT-3′) and Tif2NPR (5′-GCTCATCTGGCCTGTCATCT-3′). RNA sizes were estimated based on 26S and 18S migration.

### RT-PCR analysis of viral fusions

RNA samples were prepared in the same fashion as for Q RT-PCR analysis. cDNA was produced using Superscript III reverse transcriptase (Invitrogen, Carlsbad CA) using oligo(dT) primers. Detection of viral fusion transcripts was performed using primers specific to a unique portion of the 3′ LTR of AKV (5′-CGTGGTCTCGCTGATCCT-3′), and to the second exon of Prdm14 (5′-ATCGTGGTACTTCGGGAGTG-3′) [16]. After 30 cycles of PCR, the reactions were electrophoresed on a 1% agarose gel. Positive bands were cut out and sequenced.

## Supporting Information

Figure S1 Somatic Ecotropic Viral Integrations in *Evi32* tumors. Southern blot analysis was performed on all tumors containing *Evi32* integrations using *Sac*I and *Pvu*II digestion. Probe to detect retroviral insertions is directed towards the AKV *env* gene. Asterisks indicate rearranged bands within the *Evi32* positive tumors. Differences in unrearranged band pattern between the AKXD strain 27 and 18 mice indicate the differences in copy number of AKV between the strains. Total numbers of retroviral integrations are tabulated in Supplementary Table S1.
(5.26 MB TIF)

Figure S2 *Oct-3/4* expression in AKXD tumors. Quantitative RT-PCR was performed on AKXD tumor cDNA with or without *Evi32* insertions. First four bars from left represent *Evi32* positive tumors (27-001, 27-142, 27-186, and 18-077); all others were tested for *Evi32* rearrangements by Southern blot analysis and were negative for *Evi32* rearrangements. AB2.2 cDNA serves as a positive control for *Oct-3/4* expression. Figure shows expression of *Oct-3/4* in the majority of AKXD tumors from all lines tested.
(0.26 MB TIF)

Table S1 Summary of tumor data in *Evi32* tumors. Table summarizes the types of rearrangements observed in the B- and T-cell receptor in the *Evi32* tumors, the pathology of the tumors, the total number of insertions identified using VISA and via Southern blotting, and lastly other common sites identified through sequencing of VSTs. *(27–186 was classified as an enlarged marginal zone, not neoplastic; however, injection of cells obtained from this tumor were able to reconstitute in NOD/SCID mice indicating neoplastic features.)
(0.03 MB DOC)
