# Supplementary figures and images for: The Zinc Finger SET Domain Gene Prdm14 Is Overexpressed in Lymphoblastic Lymphomas with Retroviral Insertions at Evi32
Source: PLoS One. 2008 Nov 27;3(11):e3823. doi: 10.1371/journal.pone.0003823 (PMC2584371; doi:10.1371/journal.pone.0003823)

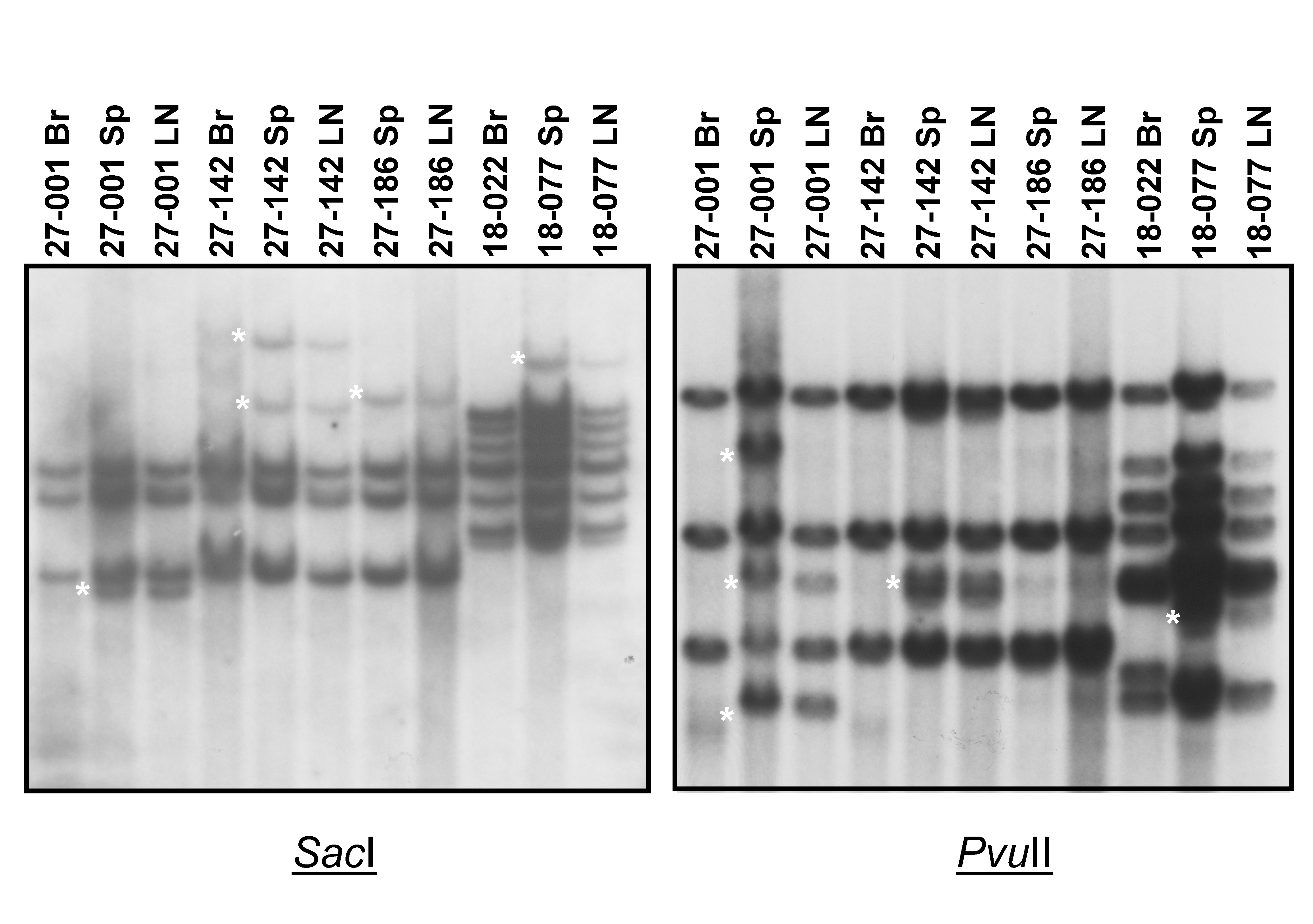

Supplement: Figure S1 — Somatic Ecotropic Viral Integrations in Evi32 tumors. Southern blot analysis was performed on all tumors containing Evi32 integrations using SacI and PvuII digestion. Probe to detect retroviral insertions is directed towards the AKV env gene. Asterisks indicate rearranged bands within the Evi32 positive tumors. Differences in unrearranged band pattern between the AKXD strain 27 and 18 mice indicate the differences in copy number of AKV between the strains. Total numbers of retroviral integrations are tabulated in Supplementary Table S1. (5.26 MB TIF) [file pone.0003823.s001.tif]

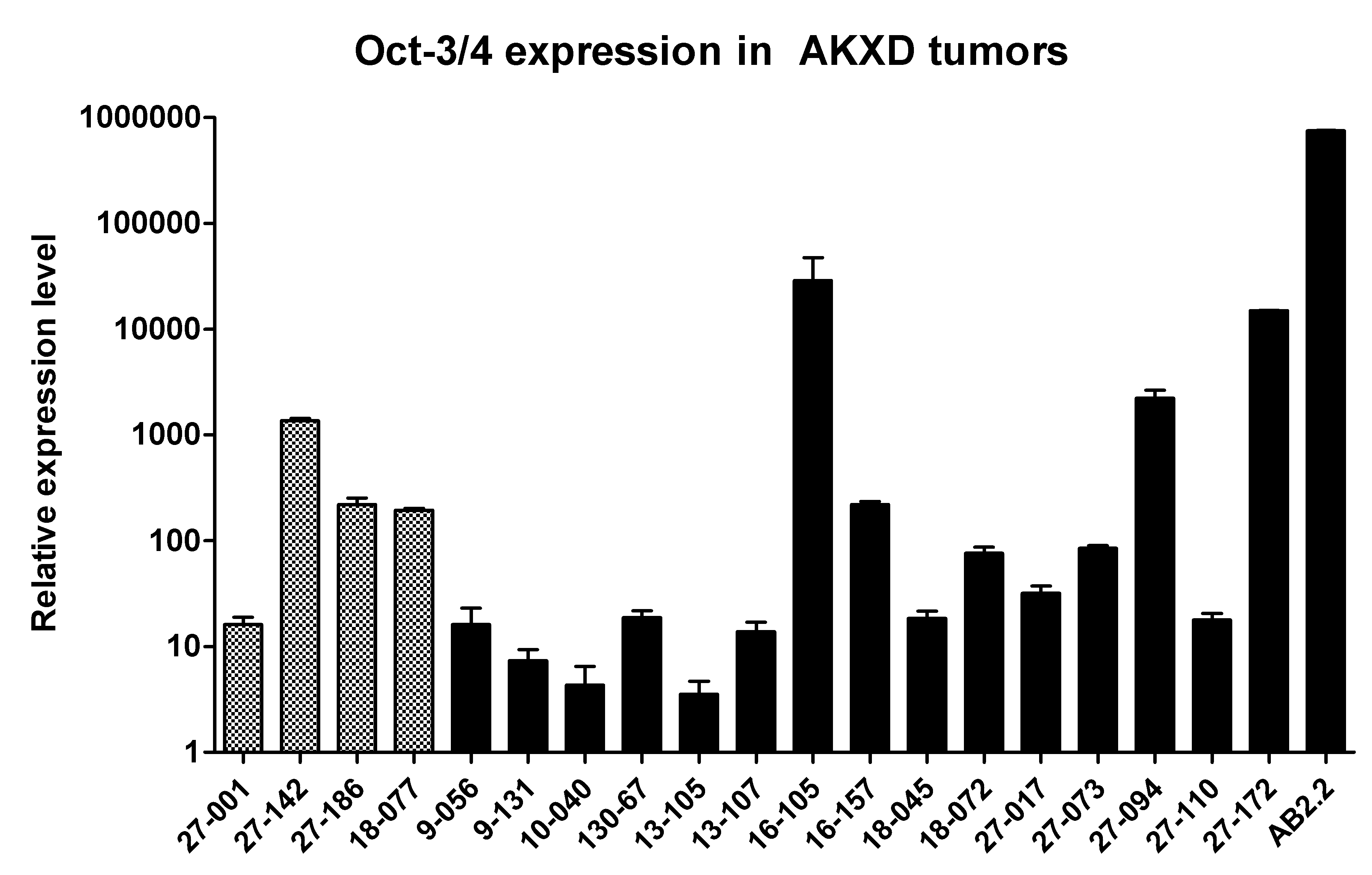

Supplement: Figure S2 — Oct-3/4 expression in AKXD tumors. Quantitative RT-PCR was performed on AKXD tumor cDNA with or without Evi32 insertions. First four bars from left represent Evi32 positive tumors (27-001, 27-142, 27-186, and 18-077); all others were tested for Evi32 rearrangements by Southern blot analysis and were negative for Evi32 rearrangements. AB2.2 cDNA serves as a positive control for Oct-3/4 expression. Figure shows expression of Oct-3/4 in the majority of AKXD tumors from all lines tested. (0.26 MB TIF) [file pone.0003823.s002.tif]
