# Supplementary material for: The Zinc Finger SET Domain Gene Prdm14 Is Overexpressed in Lymphoblastic Lymphomas with Retroviral Insertions at Evi32
Source: PLoS One. 2008 Nov 27;3(11):e3823. doi: 10.1371/journal.pone.0003823 (PMC2584371; doi:10.1371/journal.pone.0003823)
Supplement: Table S1 — Summary of tumor data in Evi32 tumors. Table summarizes the types of rearrangements observed in the B- and T-cell receptor in the Evi32 tumors, the pathology of the tumors, the total number of insertions identified using VISA and via Southern blotting, and lastly other common sites identified through sequencing of VSTs. *(27–186 was classified as an enlarged marginal zone, not neoplastic; however, injection of cells obtained from this tumor were able to reconstitute in NOD/SCID mice indicating neoplastic features.) (0.03 MB DOC) [file pone.0003823.s003.doc]

**Supplementary Table S2:**

| Animal # | Tumor Type | Histology | # of insertions by VISA | # of insertions by Southern blot | Common Sites |
| --- | --- | --- | --- | --- | --- |
| 27-001 | mixed T/B | Lymphoblastic lymphoma | 4 | 3 | - Tyrosine-protein kinase HCK - Ighmbp2 - Tcf25 |
| 27-142 | mixed T/B | Lymphoblastic lymphoma | 3 | 2 | - Auh - Commd7. |
| 27-186 | mixed T/B | Enlarged Marginal Zone* | 1 | 1 | (Fyn-Not CIS) |
| 18-077 | T-Cell | Lymphoblastic lymphoma | 0 | 1 | None |

**Summary of tumor data in *Evi32* tumors.** Table summarizes the types of rearrangements observed in the B- and T-cell receptor in the *Evi32* tumors, the pathology of the tumors, the total number of insertions identified using VISA and via Southern blotting, and lastly other common sites identified through sequencing of VSTs. *(27-186 was classified as an enlarged marginal zone, not neoplastic; however, injection of cells obtained from this tumor were able to reconstitute in NOD/SCID mice indicating neoplastic features.)
